# The role of gene variants in the pathogenesis of neurodegenerative disorders as revealed by next generation sequencing studies: a review

**DOI:** 10.1186/s40035-017-0098-0

**Published:** 2017-10-06

**Authors:** Shirley Yin-Yu Pang, Kay-Cheong Teo, Jacob Shujui Hsu, Richard Shek-Kwan Chang, Miaoxin Li, Pak-Chung Sham, Shu-Leong Ho

**Affiliations:** 1 Division of Neurology, Department of Medicine, Queen Mary Hospital, University of Hong Kong, Hong Kong, People’s Republic of China; 2 Centre for Genomic Sciences, University of Hong Kong, Hong Kong, People’s Republic of China; 3 Department of Medical Genetics, Zhongshan School of Medicine, Sun Yat-sen University, Guangzhou, People’s Republic of China; 4 Key Laboratory of Tropical Disease Control (SYSU), Ministry of Education, Guangzhou, People’s Republic of China

**Keywords:** Next generation sequencing, Neurodegenerative diseases, Genetics, Pleiotropy

## Abstract

The clinical diagnosis of neurodegenerative disorders based on phenotype is difficult in heterogeneous conditions with overlapping symptoms. It does not take into account the disease etiology or the highly variable clinical course even amongst patients diagnosed with the same disorder. The advent of next generation sequencing (NGS) has allowed for a system-wide, unbiased approach to identify all gene variants in the genome simultaneously. With the plethora of new genes being identified, genetic rather than phenotype-based classification of Mendelian diseases such as spinocerebellar ataxia (SCA), hereditary spastic paraplegia (HSP) and Charcot-Marie-Tooth disease (CMT) has become widely accepted. It has also become clear that gene variants play a role in common and predominantly sporadic neurodegenerative diseases such as Parkinson’s disease (PD) and amyotrophic lateral sclerosis (ALS). The observation of pleiotropy has emerged, with mutations in the same gene giving rise to diverse phenotypes, which further increases the complexity of phenotype-genotype correlation. Possible mechanisms of pleiotropy include different downstream effects of different mutations in the same gene, presence of modifier genes, and oligogenic inheritance. Future directions include development of bioinformatics tools and establishment of more extensive public genotype/phenotype databases to better distinguish deleterious gene variants from benign polymorphisms, translation of genetic findings into pathogenic mechanisms through in-vitro and in-vivo studies, and ultimately finding disease-modifying therapies for neurodegenerative disorders.

## Background

Traditionally, neurological disorders have been classified and diagnosed based on clinical features such as symptom-onset and disease course, and characterization of physical signs to allow localization of abnormalities in the nervous system. While most acute neurological conditions can be diagnosed with reasonable certainty, the same cannot be said to be true in chronic neurodegenerative conditions, where the definitive diagnosis can often only be ascertained by specific pathologic findings. For example, studies have shown that the accuracy of Parkinson’s disease (PD) diagnosis by neurologists was only 65–75%, which has not changed significantly in the last two decades [1].

Even in diseases that are hereditary with a clear genetic contribution, the clinical diagnosis remains difficult due to significant heterogeneity both in clinical features and genetic causes. This is evident in spinocerebellar ataxias (SCA) and hereditary spastic paraplegia (HSP). To date, 40 SCAs have been characterized, and 28 causal genes have been identified [2]. Genetically defined HSPs are assigned the symbol SPG (spastic gait) followed by a number. It is a highly heterogeneous group of disorders, with more than 80 genes or loci implicated [3]. There is significant overlap in clinical features of the two syndromes that makes diagnosis based on phenotype alone difficult. For example, patients with SCA1 may present initially with spastic paraplegia before development of cerebellar ataxia, and thus be misdiagnosed with HSP [4]. Conversely, patients with the HSP subtypes SPG4, SPG6, SPG31 and SPG37 may present with cerebellar atrophy [5].

These observations illustrate the shortcomings in the diagnosis of neurodegenerative disorders based on phenotype alone. The diagnostic criteria of these disorders are heavily reliant on clinical features, with little regard to the underlying etiology. Patients with similar clinical features due to different etiologies are often classified under the same diagnosis, resulting in a highly variable disease course and prognosis. The uncertainty in clinical diagnosis also renders recruitment of appropriate patients into research studies difficult, hindering the study of pathogenic mechanisms and the search for disease-modifying therapies.

In this review, we will discuss the role of genetic variants in hereditary and sporadic neurodegenerative disorders and the insights to etiology and pathogenic mechanisms afforded by advances in genetic sequencing and analysis. Possible mechanisms of pleitropy and future directions to help translate genetic findings to therapeutic interventions will be discussed.

## Genetics: insights into etiology

While the etiology of common, sporadic neurodegenerative disorders is likely to be multifactorial and more difficult to dissect, hereditary conditions with Mendelian inheritance offer the opportunity to study pathogenic mechanism by identification of the causal genetic mutation and characterization of its effect on function. Early success in genetic linkage studies in the 1980’s led to discovery of large numbers of genetic causes of monogenic diseases. It was discovered that patients diagnosed with one disorder based on phenotype often had many different genetic causes, as illustrated by the vast number of mutations associated with SCA and HSP. Using cell-based or animal-based studies, it became possible to define the pathogenic mechanism of a mutation, shedding light on cellular pathways involved in creating an abnormal phenotype, thus opening up therapeutic opportunity. Phenotype-genotype correlation also became possible, and classification of diseases such as SCA, HSP and Charcot-Marie Tooth disease (CMT) based on causative loci/genes has become widely accepted. Recognition of phenotype-genotype relationship also facilitates counseling on genetic testing and prognosis.

## Next generation sequencing

One limiting factor hindering the more widespread application of genetic diagnosis was the method of DNA sequencing. Published by Frederick Sanger in 1977 [6], Sanger (also known as chain-termination) sequencing has been the most commonly used DNA sequencing method and is still considered to be the gold standard [7]. However, this method is time consuming and expensive, as evidenced by the tremendous amount of time (13 years) and money (USD$2.7 billion) spent to complete the Human Genome Project [8, 9].

The arrival of next generation sequencing (NGS) platform in 2009 has resulted in a marked acceleration of the process of identifying genetic mutations. NGS offers the ability to simultaneously sequence large number of genomic regions. The whole genome can be sequenced (whole genome sequencing, or WGS); whereas when combined with techniques of targeted capture, any subset of the genome can be sequenced, an example being whole exome sequencing (WES) [10, 11]. Currently, using NGS technology, the whole human genome can be sequenced in 2 days at a cost of under USD$3000, which continues to decline (Table 1).

**Table 1:** Comparison of NGS platforms with Sanger sequencing

	WGS	WES	Sanger sequencing
Target of sequencing	Entire genome	Protein coding regions (~20,000 genes)	Region of interest limited to 1000 bp
Run time	<7 days	<7 days	hours
Advantages	-Comprehensive sequencing	-Sequencing of all protein coding regions at reduced cost compared with WGS	-High accuracy -Flexible target regions
Disadvantages	-Expensive -Large amount of data, challenging to analyze	-unable to detect mutations in non-coding regions	-time consuming and expensive for large-scale sequencing projects

**Fig. 1:**
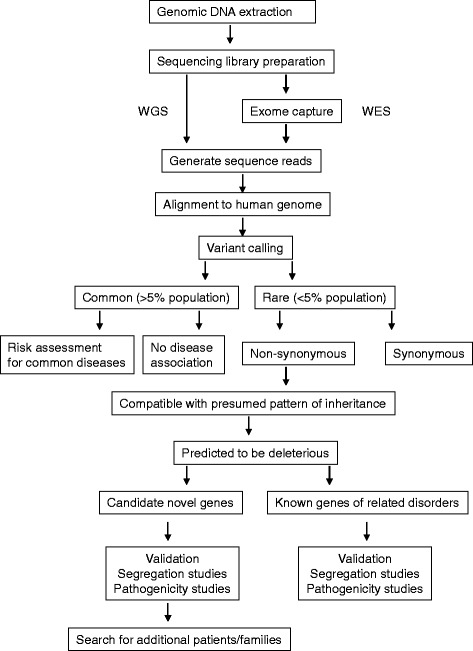
Typical workflow of NGS and bioinformatic analysis. Genomic DNA is extracted and massive parallel sequencing on various NGS platforms is performed. For whole exome sequencing, exomes capture is performed, and the raw sequences are aligned to reference genome. Variant calling is performed and filtered by various softwares. Common variants can be further analyzed for risk assessment in common diseases. Non-synonymous and rare variants can be further scrutinized according to the presumed inheritance pattern and deleteriousness predictions. Candidate variants can be confirmed by validation, segregation analyses and pathogenicity studies. For novel variants, additional affected patients and families with the same variant can further strengthen the association of the variant with disease

Data generated by NGS is analyzed using various bioinformatics tools (examples in [12, 13]). In principle, genetic variants called from NGS data are filtered and prioritized following the general strategy as outlined in Fig. 1, with the goal to select for variants that are rare, non-synonymous, compatible with the presumed inheritance pattern and predicted to be deleterious. Selected variants are then subjected to further validation by segregation analyses and pathogenicity studies (reviewed in [14]).

Before NGS, genetic mutations were identified mainly by positional cloning and linkage analysis which required large pedigrees with sufficient numbers of affected and unaffected individuals. Identification of mutations in patients with clinically and genetically heterogeneous disorders was limited by the large number of candidate genes and the associated high cost of Sanger sequencing. Moreover, in the clinical setting, diagnosis could not be established in patients with suspected underlying genetic disorders if they were not found to harbor any mutations in known causal genes. The advent of NGS changed the landscape of genetic diagnosis and offers a genome-wide, unbiased approach which weighs all genes equally, allowing all identified variants to be simultaneously assessed. WES has been advocated as the standard of care in patients presenting with heterogeneous clinical phenotypes that could result from a wide variety of genetic causes [15, 16].

## Gene variants in rare Mendelian disorders

Much of the early application and success of NGS is seen in rare Mendelian neurological disorders of high penetrance. In particular, WES, which captures all known coding exons where 85% of causal mutations are estimated to be located, has been utilized most widely. The obvious advantage of WES in clinically and genetically heterogeneous disorders is the quick identification of mutations in known genes. When no known mutation can be found, WES offers the opportunity of identifying causal mutations in novel genes.

Using NGS, at least 36 novel genes have been identified which caused CMT (reviewed in [14]). Similarly, the list of causal genes in SCA has also expanded. For example, using WES combined with linkage analysis, mutations in *TGM6* were identified in two Chinese families with SCA [17]. The importance of *TGM6* in SCA in Chinese was confirmed when WES showed a third pedigree to have a novel mutation in the same gene [18]. Two studies using WES identified mutations in the voltage-gated potassium channel encoding gene *KCND3* in families with autosomal dominant SCA, implicating reduced potassium channel function in the pathogenesis of SCA [19, 20]. Since 2012, at least 16 new genes have been identified to cause SCA (reviewed in [21]). WES was also used to identify *PMCA4* as a novel gene in a Chinese family with HSP [22]. The effect of the mutation was studied using cell-based assays which demonstrated impaired intracellular calcium extrusion, implicating dysfunction of calcium homeostasis in the development of spastic paraplegia [23].

An important role of NGS lies in its ability to uncover novel genetic causes in patients who do not harbor any known causal mutations. For example, mutations in *PMP22, GJB1, MFN2* and *MPZ* together account for about 86% of patients with CMT, with more than 50 other known genetic forms of CMT each accounting for less than 1 to 2% of patients [24]. If patients with additional associated neurological and non-neurological features are included, as many as 50% of patients with inherited peripheral neuropathy (IPN) are genetically undiagnosed [25]. Using WES in 50 families with molecularly undiagnosed IPN, 24% were found to have pathogenic or likely pathogenic variants in genes known to cause IPN, with an additional 22% harboring sequence variants of uncertain significance in IPN genes. In addition, 12% had variants in novel IPN candidate genes [26]. Another study showed that 21 of 110 index patients with IPN of unknown genetic etiology carried a sequence variant in a known CMT gene [27]. This finding was further extended in another study, which showed that 17 out of 37 (45%) families with genetically undiagnosed CMT were found to have apparent causal mutations by WES, with 3 novel candidate genes identified [28]. Similarly, in HSP where there are more than 70 genetic subtypes described, NGS techniques have helped to identify the genetic diagnosis in 240 of 519 families [29]. In SCA, 44% of French patients with autosomal dominant ataxia and 25% of Japanese familial ataxia patients remained genetically undiagnosed after SCA gene panel testing [30, 31]. Clearly, a notable proportion of patients with cerebellar ataxia have a genetic cause that remains to be identified, and NGS techniques will be invaluable in uncovering more causal genes.

## Gene variants in common and complex disorders

Many neurodegenerative disorders that are common and predominantly sporadic also have familial forms. Examples include Parkinson’s disease (PD), amyotrophic lateral sclerosis (ALS) and Alzheimer’s disease (AD). Known genetic causes of familial forms only accounted for a small proportion of patients. Just as NGS is useful in the search for novel mutations in rare Mendelian disorders, it is also invaluable in uncovering genetic causes in unexplained familial forms of these common disorders. For example, about 10% of ALS cases are familial. The four genes well known to cause familial ALS, namely *SOD1*, *TDP-43*, *FUS* and *VCP*, together only account for 50% of familial cases (reviewed in [32]). Novel mutations in *SOD1* and *VCP* were discovered through WES, as well as novel genes such as *C9orf72*, *PFN1*, *UBQLN2*. Similarly, about 5–10% of PD cases are familial, with 6 genes known to cause monogenic forms. *LRRK2* mutations, the most common genetic cause of familial PD, only account for about 10% of familial PD cases [33]. WES identified novel genes that caused familial PD such as *VPS35*, *EIF4G1* and *DNAJC6* (reviewed in [34]).

Despite the success in uncovering novel genes in familial forms, the majority of patients with these disorders are sporadic with unknown etiology, probably involving a combination of genetic and environmental risk factors. Compared with Mendelian disorders, sporadic neurodegenerative disorders are common. Genetic contribution to these disorders has therefore been postulated to be through a combination of common genetic variants, each of which increases disease risk only slightly. Genome-wide association studies (GWAS) have been used to search for association between common genetic variants and a trait. In the field of PD, GWAS have identified 28 independent risk loci [35]. However, like most GWAS hits, each of these loci individually confers only modest risk. Heritability of PD has been estimated to be about 30%, and risk loci identified by GWAS only accounted for one-tenth of this heritability [36]. Similarly, a recent GWAS estimated that single nucleotide polymorphisms (SNPs)-based heritability of ALS to be 8.5%, with genome-wide significant loci only accounting for 0.2% of that heritability [37]. A genome-wide complex trait analysis estimated the heritability of all types of ALS to be 21% [38], and twin studies showed ALS heritability to be around 61% in sporadic cases [39]. Clearly, many more heritability factors remain to be identified which could be due to risk alleles that are too rare to detect using GWAS.

Studies utilizing targeted sequencing of known causal genes in sporadic cases have shown that genetic mutations in familial cases were also found in sporadic ones. For example, genetic mutations in familial ALS genes were found in 11% of sporadic patients (reviewed in [40]). Another study reported *LRRK2* mutations in sporadic PD patients [33]. With the advent of WES, all exons can be sequenced simultaneously in large numbers of sporadic patients, thus not only enabling the search for genetic causes in sporadic cases, but also offers the opportunity to look for possibility of oligogenic inheritance in these diseases.

The concept of oligogenic inheritance in ALS was raised when some familial ALS (FALS) patients with a known causal mutation were found to harbor an additional mutation in another ALS risk gene. Using NGS techniques, a study showed that there was a significant enrichment of deleterious rare or novel alleles in ALS patients compared with controls, and that 60% of sporadic ALS (SALS) patients had rare or novel variants in 169 ALS genes [41]. With 40 candidate ALS genes, another study showed that 60.8% of SALS had at least one and 19.5% had two or more rare variants [42]. Moreover, it has been shown that increased risk variant burden is associated with earlier age of onset [43] and reduced survival [42] (Table 2). These studies showed that a significant proportion of sporadic patients harbored rare variants in ALS risk genes, and help to identify the missing heritability of ALS. These observations are also supportive of an oligogenic influence on the development and progression of ALS.

**Table 2:** Genetic contribution to the risk and progression of ALS using NGS

Study	Methods	Risk of ALS	ALS course
Couthouis et al., 2014 [41]	Targeted sequencing of 169 ALS genes in 242 sporadic ALS patients	134 novel variants were found in ALS patients versus 61 in controls. 99 rare variants were found in ALS patients versus 41 in controls. Deleterious novel and rare variants were enriched in cases vs. controls (*p* = 0.019).	APOE ε2 allele was associated with limb onset and ε4 allele was associated with earlier age of onset in limb onset ALS.
Cady et al., 2015 [43]	Targeted pooled-sample sequencing of 17 ALS genes in 391 sporadic and familial ALS patients	64.3% of familial and 27.8% of sporadic ALS patients had novel or rare variants. 3.8% of patients had variants in more than 1 ALS gene.	Patients with variants in more than 1 ALS gene had earlier disease onset by 10 years.
Pang et al., 2017 [42]	WGS of 8 familial ALS patients and WES of 46 sporadic ALS patients Variants in 40 ALS genes were examined	67% had one variant; 22% had two or more. Presence of rare variants was significantly associated with risk of ALS (*p* = 0.03). ALS patients had significantly higher rare variant burden than controls (*p* = 0.004).	FALS patients with additional rare variants had shorter survival compared with FALS patients with only one mutation. In sporadic and familial ALS, each additional rare variant increased the risk of ventilatory failure or death by 60%. Patients with two or more variants had significantly lower probability of survival than patients with zero or one variant (*p* = 0.001).

Recently, a large-scale study using WES and involving more than 7000 PD patients showed that 30% of PD patients with a known primary genetic cause had additional rare variants in established PD-related genes compared with 17% in PD patients without known mutations and 15.6% in controls. Moreover, carriers of additional variants had earlier age of onset, suggesting an oligogenic inheritance in PD [44]. Undoubtedly, with the costs of NGS continuing to decrease, further studies will uncover more rare variants to be involved in the pathogenesis of complex neurodegenerative disorders, thereby elucidating cellular pathways that may be targets for intervention.

## Pleiotropy

Just as clinically similar symptoms can be caused by a large number of genetic variants in various neurodegenerative disorders, a genetic locus can harbor variants that are associated with multiple, sometimes distinct, traits, giving rise to the concept of pleiotropy (reviewed in [45]). The mechanisms underlying pleiotropy is likely to be diverse, and understanding of these mechanisms will be crucial in our understanding of the genetic contribution and pathogenesis of neurodegenerative disorders (Table 3).

**Table 3:** Examples of pleiotropy in neurological disorders and possible mechanisms

Diverse phenotype with	Possible mechanisms	Examples	References
Different mutations in the same gene	Different mutations may have different downstream effects	*VCP* mutations are found in ALS, FTD and inclusion body myopathy.	Nalbandian et al., 2011 [47]
	Different variants confer different levels of pathogenicity	*SNCA* point mutations are highly penetrant for PD while penetrance for dosage mutation is linked to copy number; common variants increase PD risk only slightly.	Singleton et al., 2016 [36]
Same mutation in the same gene	Presence of modifier gene	*TMEM106b* minor allele is protective of FTD but not ALS in patients with *C9orf72* repeat expansion.	van Blitterswijk et al., 2014 [51]
	Oligogenic inheritance	FALS patients with *SOD1* mutation and additional rare variants in ALS genes have reduced survival.	Pang et al., 2017 [42]
	Different effects in different cell lines	*TGM6* mutation found in families with SCA and a family with leukemia.	Pan et al., 2014 [48]

### Diverse phenotype due to mutations in the same gene

Mutations in the same gene can often cause different phenotypes. For example, mutations in *CACNA1A* were described in SCA6, familial hemiplegic migraine type I and episodic ataxia type II (reviewed in [21]). Another gene, *KCND3*, previously known to cause cardiomyopathy, was also found to be implicated in autosomal dominant SCA [19, 20]. Mutations in *BSCL2* can cause different motor manifestations such as hereditary motor neuropathy, CMT2, SPG17 and has been described in a patient with rapidly progressive ALS-like phenotype [46]. *VCP* mutations are found in ALS, inclusion body myositis, HSP and CMT2 [47]. Interestingly, a *TGM6* mutation which has been implicated in SCA was found to segregate with acute myeloid leukemia [48]. Possible explanations for pleiotropy observed are: (i) different mutations in the same gene can have different downstream effects leading to different phenotypes, (ii) mutations in the same gene can have different effects in different cell lines, as exemplified by TGM6 mutation in SCA and acute leukemia.

Different phenotype may also result from different variants in the same gene which confers different levels of pathogenicity. For example, while point mutations in the α-synuclein encoding gene *SNCA* are highly penetrant and cause young-onset PD, dosage mutations have more variable penetrance depending on copy number. In contrast, common variants of *SNCA* increase risk of PD only slightly [36].

### Diverse phenotype with same mutation

Even within families with the same underlying causal mutation, there can be significant variability in phenotype in terms of symptoms, age of onset and disease course. For example, four patients from the same HSP pedigree with the same mutation in *BSCL2* had wide variation in phenotype ranging from asymptomatic with only electrophysiological abnormality to a rapidly progressive ALS-like phenotype [46]. Another example is the wide phenotypic variability observed in a three-generation Chinese pedigree with ALS due to the same *SOD1* mutation with some patients having bulbar onset and rapid progression while others survived much longer [49]. These and other similar observations have led to the search for modifier genes to explain the clinical heterogeneity observed with the same causal mutation.

One example of a modifier gene is *TMEM106b* which has been found to have modifying effect on the risk of fronto-temporal lobar degeneration with accumulation of the TAR DNA binding protein 43 (FTLD-TDP). The two most common genetic causes of FTLD-TDP are mutations in progranulin gene (*GRN*) and hexanucleotide repeat expansion in *C9orf72*. Patients carrying two copies of the minor allele of three *TMEM106b* SNPs were found to be under-represented in *GRN* mutation carriers with FTLD-TDP; in other words, homozygosity in the minor allele was protective (reviewed in [50]). Intriguingly, after the discovery of *C9orf72* repeat expansion as a genetic cause of FTLD as well as ALS with TDP-43 pathology, it was found that the minor allele in *TMEM106b* also protected against development of FTD, but not against ALS, in *C9orf72* expansion carriers [51]. Conversely, ALS patients with *C9orf72* repeat expansion and the protective *TMEM106b* alleles had better cognitive performance. While these observations confirm the modifying effect *TMEM106B* genotype has on the risk of developing FTD, they also raise the question of why there is a differential effect on the cortex and on the spinal cord, when the underlying mutation (*C9orf72* repeat expansion) and the pathology (TDP-43 aggregation) are the same.

Another possible mechanism to explain pleiotropy resulting in diverse phenotypes with the same causal mutation is oligogenic inheritance. NSG has been very successful in this regard because it confers the advantage of detecting variants in all genes simultaneously. Again using ALS as an example, it was noted that significant heterogeneity within the same kindred existed in familial ALS with *SOD1* mutation [49]. A recent study showed that within this pedigree, patients who had additional rare variants in 40 ALS genes had the most rapid disease progression and smallest chance of survival [42], raising the possibility that even with a highly penetrant causal mutation, the disease course is still modifiable by additional variants. This observation was further extended to sporadic cases, with patients harboring two or more rare variants having significantly lower survival than patients with zero or one rare variant. Another study showed that ALS patients with rare variants in more than one ALS gene had earlier age of onset [43]. These observations suggest that the development and progression of ALS likely involve a combined effect of multiple genetic variants, and clarification of pathways involving these genes will identify possible therapeutic targets for disease modification.

A recent study showed the possible oligogenic influence on PD, with about 30% of PD patients with a known causal mutation harboring additional variants [44]. Carriers of additional variants were found to have earlier age of onset. The most common causal mutation in this study was *LRRK2* G2019S, and the most common additional variant in these patients occurred in *ATP13A2*. Thus, the presence or absence of additional variants in *LRRK2* mutation carriers may account for the variable penetrance observed.

## Conclusion

NGS has revolutionized the field of genetics in neurological disorders. The list of genes responsible for neurological dysfunction is rapidly expanding, helping to fill the gap of missing heritability in many neurodegenerative disorders. Identification of a novel gene leads to exploration of a new pathway in the pathogenesis of a disease, and genetic discovery is a starting point for elucidating pathogenic mechanisms. With the ever-reducing costs of NGS, the challenges ahead will be accurate analysis and interpretation of large amount of genetic data, and translating genetic data into pathogenic mechanisms and ultimately therapeutic opportunities. So far, WES has been most widely used, but obvious limitations include the inability to detect non-coding variants in intronic regions, large fragment copy number variations and chromosome derangement. Another challenge lies in the separation of benign from disease-causing variants. It has been estimated that in the genome of a single individual, about 10,000 to 11,000 non-synonymous variants exist [52, 53]. It has also been reported that up to 27% of allegedly disease-causing variants were later found to be common benign polymorphisms [54]. Future directions include better tools to differentiate pathogenic from benign variants at the bioinformatic and functional levels, collection of more human genome data of different ethnicities, collaborative effort to establish extensive public databases, and methods to study non-coding variants, functional genomics, and epigenetics.

The observation of pleiotropy suggests that common pathological mechanisms may underlie clinically diverse neurological syndromes. Through identification of new and sometimes common genes in different disorders, it has become clear that common pathways exist in the pathogenesis of neurodegeneration: aberrant ion channel function, mitochondrial dysfunction, defects in intracellular trafficking and axonal transport, abnormal protein aggregation and clearance. Much work still needs to be done to elucidate cellular targets for intervention. With the advent of NGS, genetic diagnosis is already replacing phenotype-oriented classification of neurological disorders, and individualized treatment based on genetic findings may one day be available to treat neurodegenerative diseases.

## Abbreviations

AD: Alzheimer’s disease
ALS: Amyotrophic lateral sclerosis
CMT: Charcot-Marie Tooth
FALS: Familial ALS
FTLD-TDP: Fronto-temporal lobar degeneration with accumulation of the TAR DNA binding protein 43
GWAS: Genome-wide association study
HSP: Hereditary spastic paraplegia
IPN: Inherited peripheral neuropathy
NGS: Next generation sequencing
PD: Parkinson’s disease
SALS: Sporadic ALS
SCA: Spinocerebellar ataxia
SNP: Single nucleotide polymorphism
SPG: Spastic gait
WES: Whole exome sequencing
WGS: Whole genome sequencing
